# Delayed Diagnosis of Tuberous Sclerosis Complex: A Case Report

**DOI:** 10.7759/cureus.112466

**Published:** 2026-07-11

**Authors:** Selcen Kundak, Himmet Yalabik

**Affiliations:** 1 Department of Dermatology, İzmir Şehir Hastanesi, Sağlık Bilimleri Üniversitesi, Izmir, TUR

**Keywords:** angiomyolipoma, connective tissue nevus, facial angiofibroma, periungual fibroma, retinal astrocytic hamartoma, tuberous sclerosis complex (tsc)

## Abstract

Tuberous sclerosis complex (TSC) is a multisystem genetic disorder with variable clinical expression. We report the case of a 26-year-old woman who presented with characteristic cutaneous findings, including facial angiofibromas, periungual fibromas, and a fibrous cephalic plaque. Further evaluation revealed renal angiomyolipomas and retinal astrocytic hamartomas. Despite a history of childhood seizures, the diagnosis was delayed until adulthood. This case highlights the role of dermatological examination in identifying TSC, particularly in patients with subtle or asymptomatic systemic involvement.

## Introduction

Tuberous sclerosis complex (TSC) is a multisystem genetic disorder caused by pathogenic variants in *TSC1* or *TSC2*. Although inherited in an autosomal dominant manner, the majority of cases arise from de novo mutations, and clinical manifestations show considerable variability [1].

Cutaneous findings are among the most common and earliest manifestations of TSC and often provide important diagnostic clues [2]. The diagnosis of TSC is currently based on the 2021 International TSC Consensus Criteria, which define 11 major and six minor clinical features, several of which are mucocutaneous (e.g., facial angiofibromas, hypomelanotic macules, ungual fibromas, and connective tissue nevi), together with a genetic criterion based on identification of a pathogenic *TSC1* or *TSC2* variant. A definite diagnosis requires either two major features, one major feature with two or more minor features, or an identified pathogenic variant [3]. However, the timing and severity of systemic involvement are heterogeneous, and in some patients, particularly those with mild or atypical presentations, the diagnosis may be delayed until adulthood [3].

Here, we present a patient with characteristic cutaneous findings who was diagnosed with TSC in adulthood, highlighting the role of dermatological examination in recognizing this multisystem disorder.

## Case presentation

A 26-year-old woman presented with progressively increasing facial papules of five years’ duration, initially appearing as erythematous lesions around the nasolabial folds and gradually spreading over the entire face. Her medical history was notable for self-limited epileptic seizures between the ages of two and six years. She was not receiving any regular medication, and there was no relevant family history.

**Figure 1:**
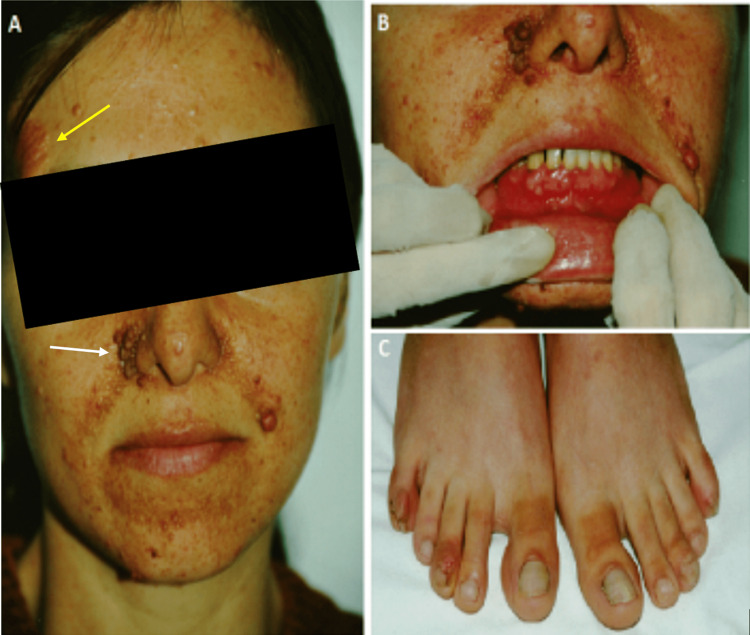
Mucocutaneous manifestations (A) Multiple skin-colored to erythematous papules distributed over the central face, consistent with facial angiofibromas (white arrow), along with a slightly elevated plaque on the frontal region compatible with a fibrous cephalic plaque (yellow arrow). (B) Multiple violaceous papules along the gingival margins, consistent with gingival fibromas. (C) Periungual fibromas (Koenen tumors) involving the toes, with associated nail plate deformity.

Dermatological examination revealed numerous well-demarcated papules (1-8 mm) distributed across the face, predominantly skin-colored with occasional erythematous or hyperpigmented features, consistent with facial angiofibromas (Figure 1A). Oral examination showed multiple violaceous papules along the gingival margins, suggestive of gingival fibromas (Figure 1B). A 1.5-cm, slightly elevated, skin-colored plaque on the right frontal region was compatible with a fibrous cephalic plaque (Figure 1A). Multiple periungual lesions were observed on both hands and feet, including a 0.7-cm lesion causing nail plate distortion, consistent with Koenen tumors (Figure 1C). In addition, three hypomelanotic macules, each measuring 0.5-1 cm in diameter, were identified on the lower extremities; hypopigmentation was confirmed on Wood's lamp examination [4]. Several hyperpigmented macules were also noted on the trunk.

**Figure 2:**
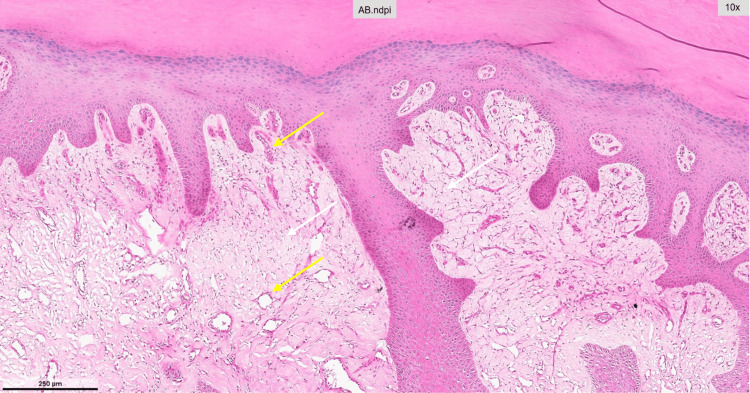
Histopathological features of facial angiofibroma Histopathological examination of a facial papule demonstrating angiofibroma. Proliferation of stellate fibroblasts within a fibrous stroma (white arrows) and ectatic blood vessels in the upper dermis (yellow arrows) are observed (H&E, ×100).

Based on these findings, TSC was suspected. Histopathological examination of excised nasolabial papules confirmed angiofibroma (Figure 2).

**Figure 3:**
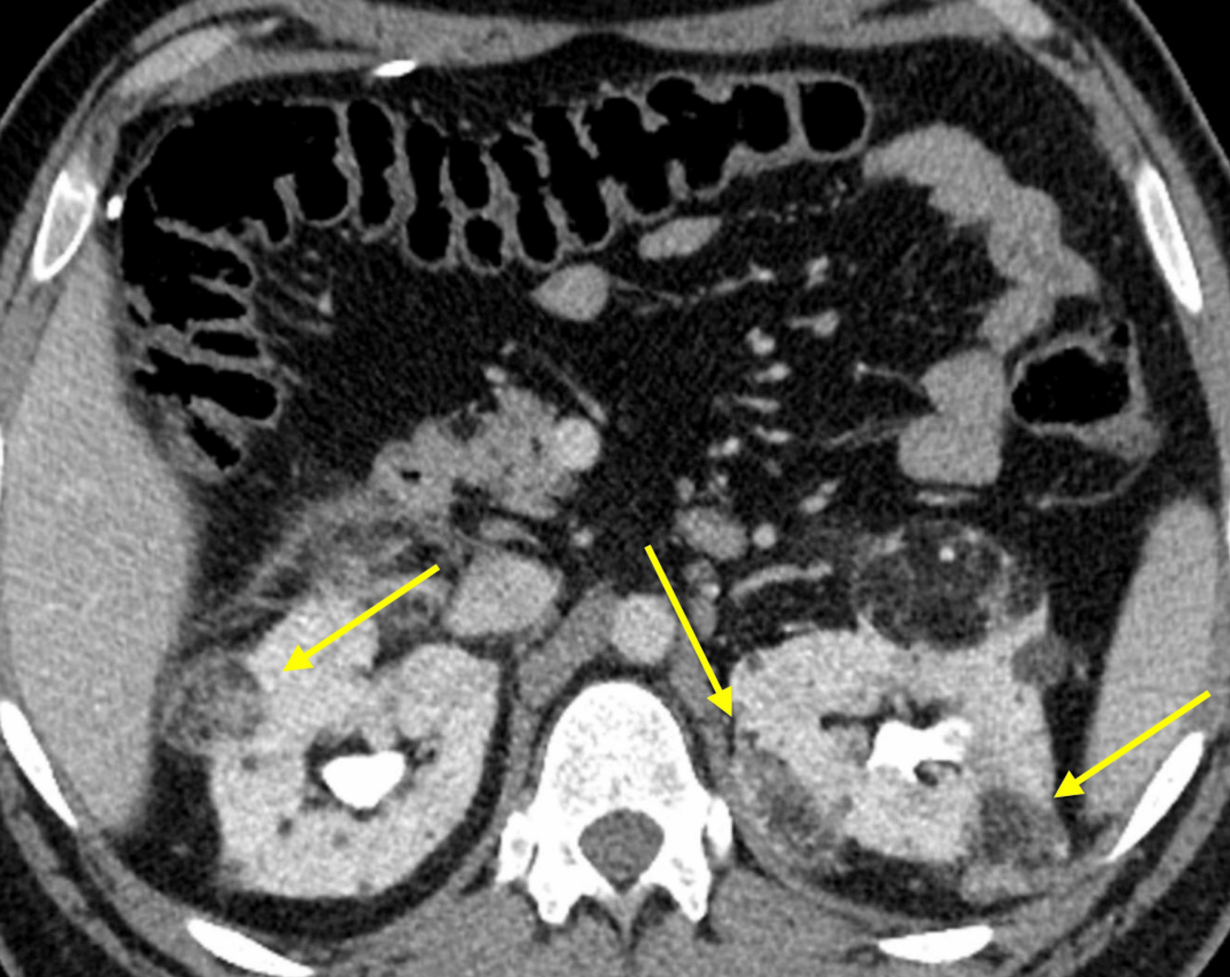
Bilateral renal angiomyolipomas on abdominal CT Axial CT images revealing multiple fat-containing hamartomatous lesions in both kidneys, indicated by yellow arrows.

Abdominal ultrasonography revealed increased echogenicity and heterogeneity in both renal regions. Contrast-enhanced computed tomography (CT) demonstrated multiple bilateral hypodense lesions with fat density, more prominent on the left, consistent with renal angiomyolipomas (Figure 3).

**Figure 4:**
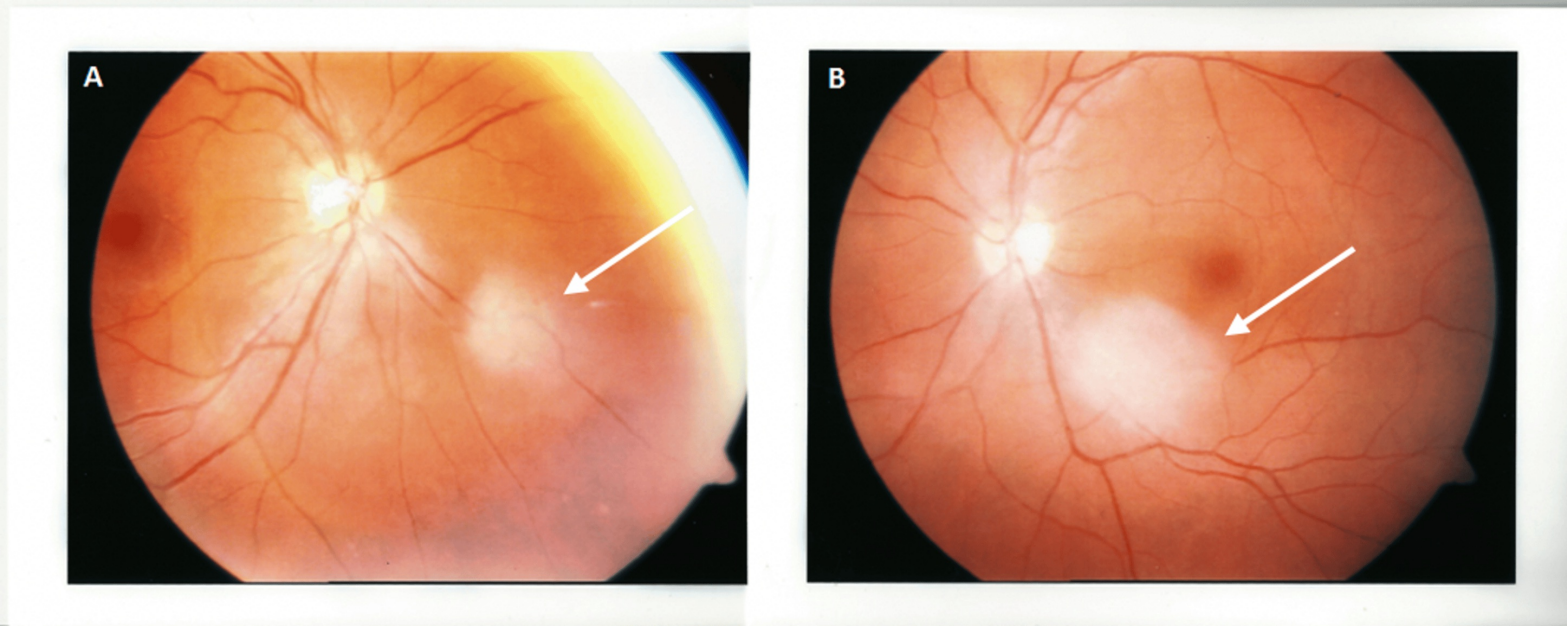
Fundus photography demonstrating non-calcified retinal astrocytic hamartomas (white arrows) (A) Lesion measuring approximately 1.5 disc diameters in the inferonasal quadrant of the right eye. (B) Lesion measuring approximately 3 disc diameters in the inferotemporal quadrant of the left eye.

Ophthalmologic evaluation with fundus photography revealed bilateral non-calcified retinal astrocytic hamartomas measuring approximately 1.5 and 3 disc diameters, respectively (Figure 4).

Further investigations, including Wechsler Adult Intelligence Scale - Fourth Edition (WAIS-IV) testing [5], echocardiography, and cranial imaging, could not be completed. No overt cognitive impairment was evident on clinical assessment. The patient declined further evaluation and was subsequently lost to follow-up.

## Discussion

TSC is a multisystem genetic disorder caused by pathogenic variants in *TSC1* or *TSC2*, resulting in constitutive activation of the mammalian target of rapamycin (mTOR) signaling pathway and the development of hamartomatous lesions in multiple organs [1,6,7]. Although inherited in an autosomal dominant manner, approximately two-thirds of cases arise from de novo mutations [7]. The clinical spectrum is remarkably heterogeneous, ranging from severe neurodevelopmental impairment with refractory epilepsy to relatively mild phenotypes that may remain unrecognized until adulthood [1,6,7].

The present case is noteworthy because the diagnosis was established at 26 years of age despite the presence of multiple major diagnostic criteria. Most patients with TSC are diagnosed during infancy or childhood, commonly following the onset of seizures, developmental delay, cardiac rhabdomyomas, or characteristic mucocutaneous findings. Davis et al. demonstrated that the majority of patients are identified within the first years of life, highlighting the importance of early clinical recognition [2]. However, delayed diagnosis remains a recognized challenge, particularly among individuals with attenuated neurological involvement and preserved cognitive function [7,8]. In our patient, childhood epilepsy resolved spontaneously without further neurological sequelae, while the cutaneous manifestations evolved gradually and became clinically prominent only during adulthood, contributing to delayed recognition.

Comparable adult-onset presentations have been reported in the literature. Domínguez-Valdez et al. described a 60-year-old woman whose childhood seizures had resolved spontaneously and who showed no intellectual deficits in adulthood; TSC was recognized only after an unrelated dermatological complaint prompted further evaluation, which revealed cutaneous stigmata, cortical tubers, renal angiomyolipomas, and a cardiac rhabdomyoma [9]. In contrast, Cruz-Zúñiga et al. reported an adult patient with a history of poorly controlled seizures and intellectual disability since childhood who remained undiagnosed until TSC was incidentally identified during pregnancy [10]. Together with the present case, these reports illustrate that adult diagnosis of TSC may occur across a broad spectrum of neurological severity, from preserved cognitive function to substantial neurodevelopmental impairment.

Cutaneous manifestations are among the most common and diagnostically informative features of TSC. According to the International TSC Consensus criteria, facial angiofibromas or fibrous cephalic plaque, hypomelanotic macules, and ungual fibromas constitute major diagnostic features [3]. In our patient, the coexistence of facial angiofibromas, periungual fibromas, gingival fibromas, fibrous cephalic plaque, and hypomelanotic macules provided strong clinical evidence supporting the diagnosis. Previous studies have emphasized the pivotal role of dermatologists in identifying TSC, particularly in patients lacking severe neurological symptoms [6,11]. This case further underscores the importance of detailed dermatological examination in adults presenting with multiple facial papules and other characteristic cutaneous findings.

An additional noteworthy aspect of our case is the relatively late progression of facial angiofibromas. These lesions are reported in approximately 75-90% of patients and typically become apparent during early childhood [6,11]. Nevertheless, substantial variability exists regarding the age of onset, number, and severity of lesions. Progressive enlargement and increasing numbers of angiofibromas during adolescence and adulthood have been reported in patients with otherwise mild disease phenotypes [6,7]. The gradual progression observed in our patient over a five-year period illustrates the broad phenotypic spectrum of TSC and highlights how slowly evolving cutaneous manifestations may delay diagnosis.

Systemic involvement in TSC is often clinically silent and may remain undetected unless actively investigated. Renal angiomyolipomas represent one of the most frequent extracutaneous manifestations and are a major source of morbidity in adult patients [1,6,7]. Bilateral and multiple angiomyolipomas, as observed in our patient, are highly suggestive of TSC and carry a risk of hemorrhage and progressive renal impairment [3,7]. Retinal astrocytic hamartomas are likewise characteristic manifestations but frequently remain asymptomatic throughout life [1,7]. The coexistence of bilateral renal angiomyolipomas and retinal astrocytic hamartomas in our patient highlights the importance of comprehensive multidisciplinary evaluation once TSC is suspected.

Overall, our patient met five major diagnostic criteria, facial angiofibromas with a coexisting fibrous cephalic plaque (a single combined criterion), ungual fibromas, hypomelanotic macules, renal angiomyolipomas, and retinal astrocytic hamartomas, along with one minor criterion, intraoral fibromas, fulfilling the threshold for a definite diagnosis of TSC by a wide margin [3]. This observation reinforces that the diagnostic delay in this case reflected the gradual evolution of cutaneous findings rather than an insufficiency of clinical criteria.

Neurological involvement remains the principal determinant of morbidity and quality of life in TSC [1,8]. Epilepsy affects the majority of patients and is strongly associated with adverse neurodevelopmental outcomes when seizure onset occurs early and remains poorly controlled [8]. However, a subset of patients follows a milder neurological course characterized by self-limited seizures and preserved intellectual functioning. Our patient belongs to this subgroup, demonstrating that the absence of significant cognitive impairment does not exclude the diagnosis and may contribute to prolonged diagnostic delay. A notable limitation of this case is that cranial MRI could not be performed, precluding assessment of cortical tubers, subependymal nodules, and subependymal giant cell astrocytomas, which are of particular relevance given the patient's history of childhood epilepsy.

Recent advances in understanding the molecular pathogenesis of TSC have led to the development of targeted therapeutic strategies. mTOR inhibitors, particularly everolimus, have demonstrated efficacy in reducing the size of renal angiomyolipomas, subependymal giant cell astrocytomas, and cutaneous angiofibromas [1,6,7]. Consequently, early diagnosis has become increasingly important, not only for surveillance of multisystem involvement but also for timely implementation of disease-modifying therapies.

## Conclusions

TSC should be considered even in adult patients presenting with characteristic cutaneous findings, especially when systemic manifestations are subtle or clinically silent. This case underscores that delayed diagnosis may occur in the absence of overt neurological impairment and reinforces the central role of dermatologists in recognizing TSC and facilitating appropriate multidisciplinary evaluation.

Current guidelines emphasize not only timely diagnosis but also structured, lifelong surveillance through a multidisciplinary approach involving dermatology, neurology, nephrology, ophthalmology, and genetic counseling, to enable early detection and management of organ involvement and to support family counseling regarding inheritance risk.
